# The Cholera in Spain

**Published:** 1860-12

**Authors:** 


*The Cholera in Spain.*—The "Siglio Medico" of Madrid contains an article which shows that the cholera has regularly broken out in some part of Spain since 1854. From the 1st of May to the 29th of June of this year, 5344 cases occurred at Malaga, the deaths being 2267. Many provinces have been invaded, but Madrid has as yet escaped.

